# Supplementary material for: Derivation and characterization of an embryonic‑derived muscle progenitor cell line from Atlantic salmon (Salmo salar)
Source: In Vitro Cell Dev Biol Anim. 2026 Sep 1;62(7):1026–40. doi: 10.1007/s11626-026-01221-8 (PMC13582070; doi:10.1007/s11626-026-01221-8)
Supplement: Supplementary file 1 [file 11626_2026_1221_MOESM1_ESM.docx]

# Supplementary Data

# Table S1

| Target Gene | Forward Primer Sequence (5’ -> 3’) | Reverse Sequence (5’ -> 3’) |
| --- | --- | --- |
| *pecam1* | CAACCCCAAGAGAGTGCGTT | AACGACAGAACCGCAGGAAG |
| *vwf* | CAACCCCAAGAGAGTGCGTT | AACGACAGAACCGCAGGAAG |
| *kdr* | GAGAGTGAACGTCAGTGCGA | TCAGAATCACACCAGAGCCG |
| *myf5* | CTCCGACAGCATGGTTGACT | ACTATGTTAGACAGGCGGGC |
| *myl1* | GCAGTATTAACTTCGAGGCCTTTGT | TGAAGTAACCGTGGGGGAGTG |
| *tmp1* | ATCGACGACTTGGAAGATGAGTT | AGGCGTCTTTGATGAGGTGTAA |
| *acta1* | CCTAATGGTTGTCAGGAGACGAA | ACATCTTGGTGGGTTCTGGTTT |
| *myod1* | TGCTCAGACGGAATGATGGAT | GGCAGTCCAAACTGGAAATAACA |
| *myog* | CCCAACCCAGGGTCTCGTC | CGTTGCTGTAGCTCTGGGTA |
| *tnnt3a* | CGAGGCGGAAGCCCAAAAG | AAAGTGAGCATCGATCAGGGC |
| *ube2l3* | GGAGGCTGCACAAGGAACTC | CTCGATCCTGAACGCACCTT |
